# An Analysis of the Motivation Mechanism of the Formation of Corporate Health Strategic Innovation Capability Based on the K-Means Algorithm

**DOI:** 10.1155/2022/3647549

**Published:** 2022-01-30

**Authors:** Tingting Shang

**Affiliations:** School of Information Management, Xinjiang University of Finance & Economics, Urumqi, Xinjiang 830000, China

## Abstract

Improving enterprises' independent innovation capability is critical to improving their competitive strength, industries' independent innovation capability, industries' international competitiveness, and countries' independent innovation capability, as well as building an innovative country. It is now the era of strategic innovation. Many growing businesses are focused on strategic innovation, but they overlook the issue of ensuring that strategic innovation is implemented. Management and innovation are perennial themes in the long-term development of businesses. The most pressing issue in enterprises' strategic innovation activities is how to combine their strategic direction with their superior ability and choose a strategic innovation path that is appropriate for their development. This paper uses data mining theory to establish the K-means clustering algorithm to identify the best strategic orientation of enterprise innovation strategic direction selection based on the existing advantages and capabilities of enterprises based on the analysis of the direction of enterprise strategic innovation.

## 1. Introduction

Nowadays, society is developing rapidly and the competitive environment is fierce. The key to how enterprises can grow reversely in this environment lies in innovation. From the enterprise level, the creative ability of Chinese enterprises is not high, and the subject status is difficult to establish. The innovation power is insufficient, and the institutional foundation and social and cultural foundation are still relatively weak [1]. There is a shortage of external and internal investment resources for innovation, and a market-oriented enterprise's independent innovation mechanism has yet to be formed. In order to gradually solve these problems, we must form a linkage mechanism between the government, industry associations, and enterprises and establish an institutional system and a policy system to promote enterprises' independent innovation [2]. Management innovation is driven by the internal and external motivation of enterprises. A specific analysis of the motivation of management innovation can enable us to have a scientific understanding of the causes and laws of management innovation so as to provide guidance for future management innovation activities [3]. From the perspective of enterprise innovation ability, enterprises must have the ability to ensure that innovation activities are carried out in the established direction, and the ability is reflected by a series of ability elements [4].

In the face of intense market competition, businesses have been put to the test in terms of both survival and development. As a result, businesses must innovate [5]. The majority of entrepreneurs believe that technology is at the center of innovation. They believe that the only way for businesses to increase profits and gain competitive advantages is through technological reform and innovation. In fact, without a strategy, innovation will fail [6]. In this era of technological homogeneity, strategic innovation can help businesses create more value. Only strategic innovation can truly realize an enterprise's value creation. Breaking the original survival strategy, paying attention to operating enterprises with new strategic thinking, and constantly identifying and cultivating strategic innovation ability are all things that businesses must do. Only in this way will we be able to effectively implement strategic innovation and, as a result, create a continuous competitive advantage, allowing businesses to grow sustainably in the market [7]. When a company discovers that its strategy is no longer generating value for customers, it has the ability to reidentify, acquire, and integrate the industry's internal and external environmental resources. The current profit situation is changed using the strategic innovation method, completing the enterprise's strategic innovation goal. It is based on the accumulation of knowledge and is linked by organizational learning. Its goal is to continuously add value to businesses and customers in order to ensure that businesses have a long-term competitive advantage in the market. The K-means algorithm is used to build a clustering mining model in this paper. It has been successfully used in practice to evaluate mining results, form knowledge, and promote the development of a company's strategic innovation capability.

Innovation is the soul of a nation's progress and the inexhaustible motive force of a country's prosperity. Nowadays, innovation has increasingly become an important symbol of the liberation and development of social productive forces, which is related to the development process of a nation. Management and innovation are the eternal themes of the sustainable development of enterprises [8]. Management innovation mainly includes management idea innovation, organization innovation, management method innovation, system innovation, and cultural innovation. In the original sense of innovation, technological innovation is the commercial application of scientific and technological achievements, and this process should be mainly completed by enterprises. It is not only the main body of the market economy but also the main body of technological innovation [9]. The strength of a country's independent innovation capability is mainly determined by enterprises, and the key to improving the independent innovation capability is to improve the independent innovation capability of enterprises [10]. Based on the analysis of the dynamic mechanism of enterprise strategic innovation capability formation and the K-means algorithm, this paper constructs and designs a clustering mining model of enterprise strategic innovation capability based on the K-means algorithm. The K-means algorithm has high computational performance. It is verified that the algorithm is suitable for the research of this paper, which can better reflect the characteristics of the data in this paper, and the model is successfully applied to the analysis of strategic innovation capability of enterprises.

## 2. Related Work

According to literature [11], trust, integration, and collaboration are three-dimensional mechanisms for enterprises to realize integrated innovation. When conducting independent innovation, literature [12] suggests that businesses should invest heavily in technology research and development. Empirical evidence supports the positive relationship between R&D spending and independent innovation. From the perspective of absorptive capacity, literature [13] studies believe that good technical resources and a strong capability foundation are prerequisites for enterprises to engage in integrated innovation and achieve technological leapfrogging. Endogenous innovation, as opposed to imitation innovation, external introduction, and cracking, is a spontaneous behavior in the system, according to literature [14]. Literature [15] investigates the technology-driven formation mechanism of integrated innovation capability and develops a theoretical framework for the action mechanism of enterprise technology integration capability based on the three dimensions of technology integration. From various perspectives, literature [16] discusses and analyzes the application process and some application status of data mining in E-government. According to literature [17], technology integration is a process of innovation based on an enterprise's existing technological processes and capabilities. Literature [18] introduces four analytical frameworks: global technology framework, organizational framework, active learning, and technology transfer at the enterprise level. Literature [19] puts forward that independent innovation is the breakthrough of technology explored by enterprises through their own efforts or joint research. Literature [20] introduced the idea of ontology to build a government data warehouse. The successful construction of enterprises' basic information data warehouse verified the feasibility of the construction method of the government data warehouse. Literature [21] provides a possible attempt to explain the changes of endogenous technology. Literature [22] established a long-term economic growth model based on innovation. Literature [23] puts forward endogenous innovation and imitation innovation in parallel when analyzing economic growth. Reference [24, 25] designed an implementation scheme of the system based on multilevel architecture. When analyzing the change process of European science and technology policy, literature [26] proposed an integrated innovation policy integrating “scientific innovation policy” and “industrial innovation policy.” This view is actually an extension of the concept of the regional innovation system and the national innovation system. Literature [27] puts forward that independent innovation is an activity for innovation subjects to achieve scientific and technological breakthroughs by relying on their own strength so as to support and lead economic and social development and ensure national security. Literature [28] first incorporated technological progress into the economic growth model, analyzed the internal factors, and internalized a part of the role of technological progress. In the enterprise strategic innovation model studied in this paper, enterprises innovate on the basis of their own inherent strategies. Through the K-means algorithm, enterprises can find the best way for enterprise innovation, and through repeated iterations, they can find out the strategic innovation points gradually clear in the process of clustering so as to determine the optimal direction of enterprise strategic innovation.

## 3. Methodology

### 3.1. The Formation Mechanism of the Enterprise's Integrated Innovation Capability

A new idea, a new discovery, a new organizational form, a policy system, and an institutional framework are all examples of innovation. Although this is a broad definition, innovation in the economic sense is very specific. It refers to a commercially motivated production process that aims to capture or maintain market share and maximize profits. With society's rapid development, competition has never been more fierce. It is critical for developing businesses to recognize that, in the face of fierce social competition, their strategies must be adjusted or recustomized [29].

**Figure 1:**
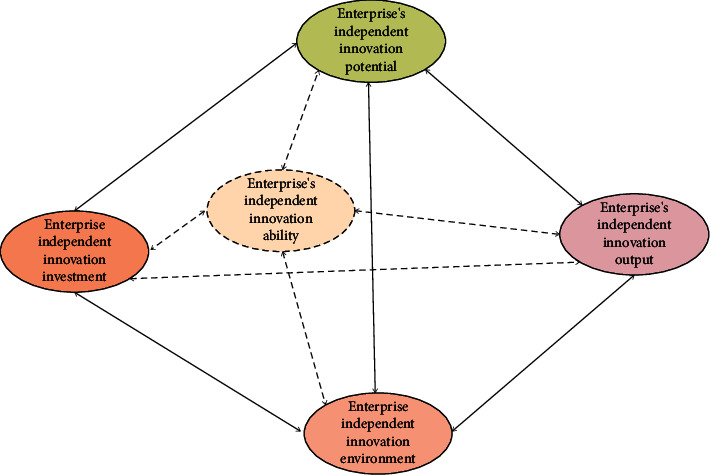
Enterprise's independent innovation capability system.

What is the strategic innovation capability of enterprises? It is considered that with the goal of continuously creating enterprise and customer value, in the process of successfully completing the cognition, formulation, implementation, and control of enterprise innovation strategy, the existing resources inside and outside the enterprise can be discovered, acquired, utilized, and integrated to improve the utilization rate of existing resources or create new resources. In this way, the system composed of various elements of strategic innovation ability needed by enterprises to gain sustainable market competitive advantage can be ensured. The independent innovation capability system of enterprises is shown in Figure 1.

The research on integrated innovation capability mainly focuses on its formation mechanism. Integrated innovation is a process in which the innovation subject optimizes and reorganizes the elements to form an organic whole with multiple functions and adaptability to evolution. Any enterprise system is not isolated and static. They are closely related to the external environment and change with the changes of the external environment to maintain their own balance [30]. It includes integration dimensions such as technology, strategy, knowledge, and organization. Different dimensions do not exist in isolation, but are interconnected and interdependent. The nonlinear interaction between various elements within each enterprise system forms a balanced system and ensures the dynamic mechanism.

**Figure 2:**
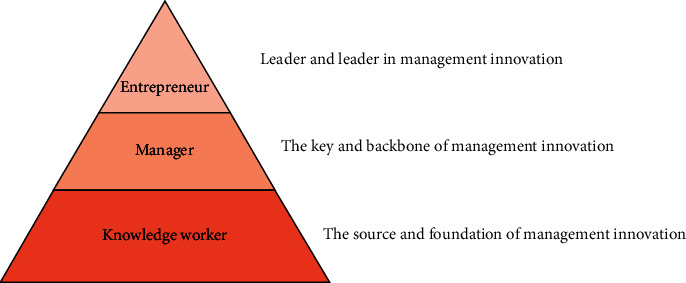
The relationship between innovation entities.

The key to establishing a management innovation mechanism is to understand the fundamental elements of management innovation. Understanding management innovation mechanisms requires a thorough understanding of the subject of innovation, as well as innovation motivation, ability, and behavior. Entrepreneurs, managers, and employees with knowledge reserves are among the subjects of innovation. Entrepreneurs are at the forefront of managerial innovation. Its primary responsibility is to coordinate, manage, and guide the innovation activities of the subject and object of innovation. The key to management innovation is managers. The source and foundation of management innovation are knowledge workers.  Figure 2 depicts the relationship between enterprise innovation and other topics.

Only with certain ability can the subject of enterprise management innovation complete the process of management innovation. This necessary ability is a synthesis of various abilities, mainly including innovation ability, transformation ability, organization and coordination ability, and learning ability. Under the action of linearity, there will be no way for enterprises' strategic innovation capabilities to correlate with each other, which will lead to the failure of functional coupling of innovation subjects. What is more serious is that the strategic innovation capability system cannot update and evolve, and the capability cannot be improved, which will eventually lead to the collapse of enterprises.

### 3.2. Evaluation Index of Creative Ability of Enterprises

**Figure 3:**
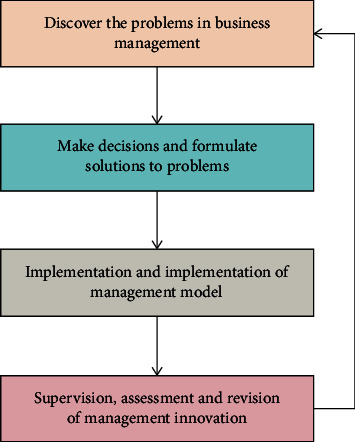
Process of management innovation.

Enterprise strategic innovation capability is a capability system aiming at providing strategic innovation. After selecting, analyzing, and distinguishing the ability factors, it finally forms organic polymerization. In this way, we can help enterprises to have the competitiveness of sustained advantages. Among them, the degree of coordination among various factors will affect the efficiency of the overall strategic innovation capability system. Therefore, enterprises must find suitable strategic innovation capabilities and implement them to adapt to the market environment. The supervision, examination, and revision of management innovation are the last stages of the process of enterprise management innovation. It refers to a series of activities to evaluate and scientifically summarize innovation achievements after a period of relatively stable operation. And it is an important link to promote enterprises to carry out a new round of innovation and bring the innovation achievements of enterprise management into play. The general process of management innovation is shown in Figure 3.

The establishment of an evaluation index system for an enterprise's creative ability is critical both in the theoretical research of independent enterprise innovation and in the practice of promoting the formation and development of an enterprise's creative ability. From the standpoint of the strategic innovation process and strategic innovation obstacles, companies must use their innovation capabilities as a foundation to ensure that the strategic innovation process runs smoothly and that strategic innovation obstacles are overcome. Organizational structure innovation, business process innovation, functional management innovation, and strategic management innovation are the different levels of management innovation. Each level of innovation has a distinct focus and set of procedures to follow. The evaluation index system of enterprises' independent innovation capability includes 4 first-level indicators. The first is the index of potential technological innovation resources, the second is the evaluation index of technological innovation activities, the third is the index of technological innovation output capacity, and the fourth is the index of technological innovation environment. There are two media between the external environment and the system. One is the boundary between the system and environment, and the other is the boundary domain between the system and environment. System boundaries absorb and release capacity, substances, and information to hinder the filtering of these contents. In this process, the boundary domain plays a buffer role in the communication between the system and the environment.

With the emergence of information technology, the shortening of the product life cycle, product diversification, and the emergence of a large number of competitors, enterprises must timely adjust the organizational structure in order to seek survival and development in the market environment characterized by variability, uncertainty, and globalization. The evaluation of enterprises' creative ability should reflect the principles of scientificity, comprehensiveness and generality, feasibility and operability, representativeness and simplicity, and comparability and reliability. The enterprise strategic innovation ability itself is helping enterprises obtain a long-term competitive advantage. It explores what kind of ability enterprises should have from the perspective of enterprise strategic innovation so as to survive in a dynamic environment, seek long-term development, and form a sustainable competitive advantage.

The evaluation of the creative ability of enterprises is an important way for enterprises to know their own creative ability. The research on the evaluation of independent innovation capability of enterprises can not only discover the regular problems in the cultivation and improvement of independent innovation capability of enterprises but also provide reasonable evaluation tools for enterprises.

### 3.3. Enterprise Strategic Innovation Based on the K-Means Algorithm

K-means algorithm is one of the classical clustering algorithms, also known as K-means. The K-means clustering algorithm is the most traditional and classic clustering algorithm at present. By constantly adjusting and updating, it integrates the mean values of adjacent data from the central point and clusters the data. Its core idea is to find out a center point so as to minimize the deviation of each data point from its nearest center point, thus forming a “class.” The algorithm has the advantages of high speed, good stability, and simple principle and is widely used in scientific research, industry and commerce, and other fields.

The K-means algorithm is based on the principle of minimizing the clustering performance index, and the commonly used clustering criterion function is the sum of the squares of the errors of each sample point in the dataset and minimizes it. The basic idea is that, firstly, *k* objects are randomly selected from *n* data objects as initial clustering centers; for that remaining other objects, according to their similarity with these cluster centers, they are, respectively, assigned to the cluster most similar to them; then, the cluster center of each new cluster is calculated; this process is repeated until the standard measure function begins to converge. The commonly used criterion function is the sum of the squared error (SSE). It is defined as follows:

(1) $$\begin{array}{c} \text{SSE}={\sum_{i=1}^{k}\sum_{p\in c_{j}}\left\|p-m_{i}\right\|}^{2}. \end{array}$$

Here, *E* is the sum of squared errors of all objects in the mining dataset, and *p* is a certain point in the space, which represents a given data object. *m*_*i*_ is the average value of cluster *C*. This criterion makes the generated clusters as compact as possible, and the clusters are separated as much as possible.

In the K-means algorithm, proximity, centroid, and objective function are the three key points of the algorithm. Usually, the proximity function is the squared Euclidean distance, the centroid is the mean, and the Euclidean distance calculation formula is as follows:

(2) $$\begin{array}{c} d_{2}^{2}={\left|x_{i1}-x_{j1}\right|}^{2}+{\left|x_{i2}-x_{j2}\right|}^{2}+\ldots +{\left|x_{ip}-x_{jp}\right|}^{2}. \end{array}$$

The selection of the initial cluster center has a greater impact on the clustering results. If the selection is not good, effective clustering results will not be obtained. We can compare the final calculation results by setting some different initial values. If the results have been stable, the selection is appropriate, but it is a waste of resources and time-consuming. Generally, the mean square error is used as the standard measurement function. The *k* clusters have the following characteristics: each cluster itself is as compact as possible, and each cluster is separated as much as possible.

The description object of cluster analysis can be expressed by a set of specific characteristics. The specific characteristic value can be a text, a numerical value, or a mixture of the two. For a given set of *n* objects, the possible ways to divide it into *k* clusters are

(3) $$\begin{array}{c} N\left(n,k\right)=\frac{1}{k!}{\sum_{j=0}^{k}\left(-1\right)}^{j}\left(\begin{array}{c} k\\ j \end{array}\right){\left(k-j\right)}^{n}. \end{array}$$

Therefore, *n* objects are divided into *k* clusters, and the number of possible cluster divisions is

(4) $$\begin{array}{c} \sum_{k=1}^{n}N\left(n,k\right). \end{array}$$

The strategic convergence emphasizes that the strategic innovation capability of enterprises is a nonlinear organic aggregation through analyzing, selecting, and classifying various enterprise capability elements so as to help enterprises gain sustainable competitive advantages.

There are *n* objects to participate in the evaluation, and there are *m* quantitative evaluation indicators. The undetermined weight coefficient of each indicator is *b*_*j*_(*j*=1,2,3,…, *m*), the original data of each object on each indicator is *X*_*ij*_(*i*=1,2,3,…, *n*; *j*=1,2,3,…, *m*), and the standard data is *U*_*ij*_(*i*=1,2,3,…, *n*; *j*=1,2,3,…, *m*). Assuming that the data corresponding to the conversion of *U*_*ij*_ is *Z*_*ij*_, the comprehensive evaluation index *y*_*i*_ for each object is

(5) $$\begin{array}{c} y_{i}=\sum_{j=1}^{m}b_{j}\cdot Z_{ij},\;i=1,2,3,\ldots ,n. \end{array}$$

According to the size of *y*_*i*_, the ranking of the comprehensive evaluation of each evaluation object can be given. The specific steps are as follows:

(1) Convert from standard data *U*_*ij*_ to *Z*_*ij*_. The conversion formula is
  (6) $$\begin{array}{c} Z_{ij}=\frac{x_{ij}-x_{j}}{S_{j}}. \end{array}$$
Among them,
  (7) $$\begin{array}{c} \bar{x_{j}}=\sum_{i=1}^{n}x_{ij},\\ S_{j}=\sqrt{\frac{\sum_{i=1}^{n}{\left(x_{ij}-\bar{x_{j}}\right)}^{2}}{n}}. \end{array}$$
(2) Determination of index weight coefficient: the criterion for determining the weight coefficient *b*_*j*_ is to make *y*_*i*_ as dispersed as possible. Even if the sample variance *σ*^2^ is the largest, the sum of squares of *b*_*j*_ is 1 at the same time. Such a set of weight coefficients is exactly the eigenvector corresponding to the largest eigenvalue of the matrix *W* = *ZT·Z*. Iti is denoted as *B*=[*b*_*m*_]. Among them,
  (8) $$\begin{array}{c} \sigma^{2}=\sum_{i=1}^{n}\frac{{\left(y_{i}-\bar{y}\right)}^{2}}{n}\;Z=\left[z_{ij}\right]=\left[\begin{array}{c} z_{11}\ldots z_{1m}\\ \ldots \\ z_{n1}\ldots z_{nm} \end{array}\right]. \end{array}$$

People are interested in using data mining methods to discover potential information and data patterns from large amounts of data, which have become a common demand. With the advancement of data mining, new mining methods emerge one after the other, the most basic of which is clustering. Among the clustering methods, the K-means algorithm is one of the most well-known and widely used partitioning methods. Cluster analysis is extremely useful in a wide range of situations. The K-means algorithm clustering result is better, and it can fully reflect the data's basic characteristics. Targeted management can be accomplished by analyzing system data. The algorithm played an important role in it and is very practical.

## 4. Result Analysis and Discussion

**Figure 4:**
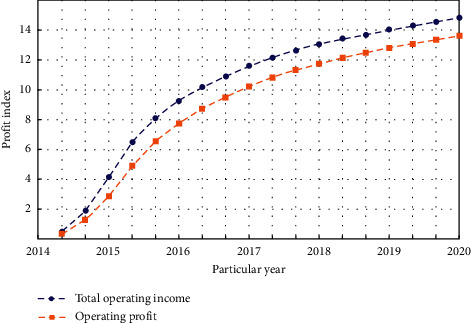
Operating performance.

The level of enterprise management determines the survival of an enterprise to a certain extent. If an enterprise wants to survive and develop in a positive direction, it must strengthen management and improve its modernization level. Corporate strategic innovation capabilities are formed in the process of corporate strategic innovation, and the targeted discovery, acquisition, utilization, creation, and integration of their strategic innovation resources are emphasized. Fund management is the top priority of enterprise management. However, a considerable number of group companies have the problem of loose fund management due to the limitation of management modes and methods. The operating performance of a company from 2014 to 2020 is shown in Figure 4.

**Figure 5:**
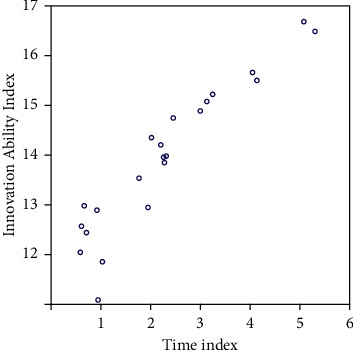
The evolution of independent innovation capabilities of enterprises.

Enterprises' creative ability means that, over a period of time, in order to cultivate their own technical ability and market competitive advantage, they achieve major breakthroughs in key industrial technologies and cultivate their own brands by effectively integrating and applying internal and external resources, in order to master or affect the basic quality of the value distribution process. Some basic information about business development can be found in the enterprise's basic information base. The amount of information in an enterprise's basic information base is growing in tandem with the economy and the enterprise itself. The overall plan made by an enterprise for its long-term survival and development on the basis of analysis and research of the external environment and internal resource conditions is known as strategy. The process of an enterprise's independent innovation capability evolving from cultivation to promotion to a new level is referred to as the “evolution of independent innovation capability.” Realize the improvement of the overall level of enterprises' creative ability and make enterprises' creative ability develop to a higher level on the basis of fully cultivating enterprises' creative ability.  Figure 5 depicts the evolution of enterprise-wide innovation capability.

**Figure 6:**
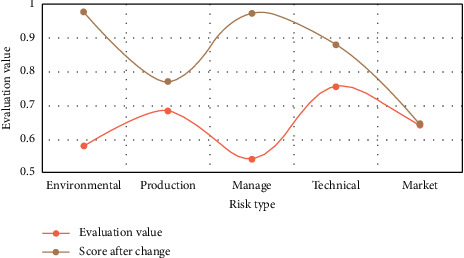
Enterprise risk evaluation.

The study of management innovation mechanisms concentrates on the components of management innovation and their interactions. Enterprises that are arbitrarily closed and have no communication with external environmental resources are in the midst of a structural organization disintegration crisis and will eventually be isolated and destroyed. Cluster ecology is an important aspect of a company's ability to innovate strategically. This feature can help businesses transform and use resources more effectively, formulate new strategies more quickly, and take advantage of opportunities to implement enterprise strategic innovation. It is not only innovation essential for social and economic development, but it is also essential for business success.  Figure 6 depicts the enterprise risk assessment.

**Figure 7:**
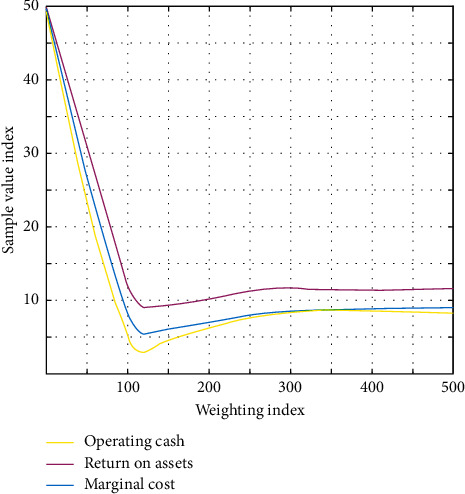
The relationship between financial risk weight and evaluation value.

For most of the risks, companies need to take certain actions to transfer or diversify the risks to reach an acceptable level for the company. The relationship between financial risk weight and sample evaluation is shown in Figure 7.

**Figure 8:**
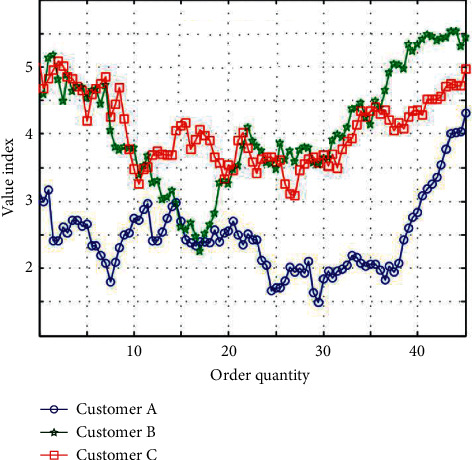
Distribution map of customer value.

System evolution emphasizes that the strategic innovation capability of enterprises will go through a dynamic process from generation, cultivation, and promotion to maintenance with the passage of time so that enterprises can make better use of the environment to evolve their own capability systems and achieve their strategic goals. The preliminarily processed data are normalized. During this period, the three center points are constantly adjusted, and after three operations with the K-means algorithm, the customer value distribution map is obtained, as shown in Figure 8.

**Figure 9:**
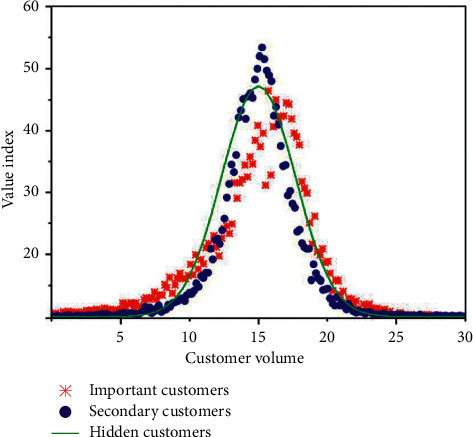
Clustering effect diagram based on K-means.

Promoting the development of enterprises with innovation is the latest topic that enterprises are facing today, and the key to the success of management innovation is to form an effective management innovation mechanism. Clustering is a common phenomenon in real society. Using the optimized K-means algorithm, customers are divided into three parts. The red part is the most important customer, the blue part is the next most important customer, and the black part is the implied customer. From the graph, we can not only intuitively see the distribution of customer value but also get the number of customers in each value range, and the clustering effect is clear. The clustering effect diagram is shown in Figure 9.

Choosing an enterprise strategy is the primary choice of enterprise management innovation. Strategy is the soul of an enterprise. If an enterprise has no strategy, it will drift with the tide and always be in a passive position. The construction of the enterprise's creative ability evaluation index system includes the determination of element items and index items. It is inseparable from the definition of enterprise's creative ability. A correct understanding of the essence of enterprise's creative ability is the basic premise for the accurate evaluation of an enterprise's creative ability. Strategic innovation capability is a capability based on strategic innovation, which is a complex of strategic innovation and enterprise capability. Innovation guides the development direction, determines the development goal, and provides the development path for enterprise capabilities. Using the K-means algorithm in this paper, enterprises can find the best way for enterprise innovation, find out the strategic innovation points gradually clear in the process of clustering, and then determine the optimal direction of enterprise strategic innovation.

## 5. Conclusions

Enterprises must constantly carry out management innovation in order to survive and develop in the fierce market competition. Enterprises can only have flexibility and superiority in management mechanisms by implementing management innovation and thus remain invincible in the competition. All innovation must be strategic, and we must seize the strategic opportunity, highlighting the significance of strategic innovation. Strategic innovation underpins technological innovation. If a company has innovative technology but it does not fit into the company's overall innovation strategy, the company's technological innovation is pointless. The strategic innovation capability of a company cannot be created out of thin air, nor can it be born within a company. Through a dynamic mechanism, an enterprise's strategic innovation capability is formed during the process of strategic innovation. The openness, being far from equilibrium, nonlinear function, and fluctuation functions of the enterprise strategic innovation capability system cause the system information to self-replicate, stabilize, and generate a new order. The K-means algorithm studied in this paper can find the most suitable way for enterprise innovation, and through repeated iterations, find out the strategic innovation points gradually clear in the process of clustering. It can determine the optimal direction of enterprise strategic innovation.

## Data Availability

The data used to support the findings of this study are included within the article.
